# Concomitant familial hypocalciuric hypercalcemia and single parathyroid adenoma: a case report

**DOI:** 10.1186/s13256-021-03051-6

**Published:** 2021-09-24

**Authors:** Simone Diedrichsen Marstrand, Charlotte Landbo Tofteng, Anne Jarløv, Line Borgwardt, Peter Schwarz

**Affiliations:** 1 Department of Endocrinology & Diabetes and Bone-metabolic Research Unit, Rigshospitalet, Copenhagen, Denmark; 2 Department of Medicine, Zealand University Hospital, Køge, Denmark; 3 Center for Genomic Medicine, Copenhagen University Hospital, Rigshospitalet, Copenhagen, Denmark; 4 Faculty of Health Sciences, University of Copenhagen, Copenhagen, Denmark; 5 Department of Endocrinology, Rigshospitalet, Blegdamsvej 9, 2100 Copenhagen, Denmark

**Keywords:** Primary hyperparathyroidism, FHH, MEN1

## Abstract

**Background:**

Primary hyperparathyroidism (PHPT) is a common endocrine disorder and the most frequent benign cause of hypercalcemia. PHPT is characterized by autonomous hypersecretion of parathyroid hormone (PTH), regardless of serum calcium levels. Familial hypocalciuric hypercalcemia (FHH) is a rare, benign syndrome only affecting the regulation of calcium metabolism. FHH is an autosomal-dominant genetic disease with high penetrance, caused by an inactivating variant in the *CASR* gene encoding the calcium-sensing receptor (CaSR). We present a unique case of concomitant PHPT and FHH without clinically actionable variants in *MEN1*.

**Case presentation:**

A 47-year-old Caucasian man with severe hypercalcemia, genetic FHH, and initially normal parathyroid scintigraphy was referred for endocrine evaluation due to nonspecific symptoms. Biochemical evaluation showed elevated serum ionized calcium and PTH. The calcium–creatinine clearance ratio was low. All other biochemical measures were normal, including kidney function. Genetic evaluation was redone and confirmed FHH. A new parathyroid scintigraphy showed a significant single adenoma corresponding to the lower left gland. The patient underwent parathyroidectomy, and a parathyroid adenoma was removed. A reduced level of hypercalcemia persisted due to FHH.

**Conclusions:**

The correct diagnosis of the underlying cause of hypercalcemia is important to ensure the right treatment. Patients with FHH should avoid operative treatment, and PHPT should be differentiated from MEN1 to determine whether surgery should include parathyroidectomy with removal of one adenoma or 3.5 hyperplastic parathyroid glands.

## Background

Primary hyperparathyroidism (PHPT) is a common endocrine disorder and is the most frequent benign cause of hypercalcemia. It is characterized by autonomous hypersecretion of parathyroid hormone (PTH), independently of serum calcium levels. Parathyroid adenoma(s) and parathyroid hyperplasia are the main causes of PHPT [1]. Familiar forms of PHPT (FPHPT) represent less than 5% of the total PHPT cases and include, among others, familial hypocalciuric hypercalcemia (FHH) and multiple endocrine neoplasia types 1 and 2A (MEN1 and MEN2A) [2].

FHH is a rare, benign syndrome only affecting the regulation of calcium metabolism, as first reported in 1972 by Foley *et al*. [3]. The familiar form FHH is an autosomal-dominant genetic disease with high penetrance, caused by an inactivating variant in the *CASR* gene encoding the calcium-sensing receptor (CaSR), first reported by Pollack *et al*. in 1993 [4]. Since then, other pathogenic variants in *GNA11* and *AP2S1* have been introduced in clinical practice. The pathogenic variants lead to lifelong hypercalcemia, with no other organ involvement. Other FPHPTs are frequently associated with other endocrine, proliferative, and/or functional disorders, such as non-endocrine tumors associated with MEN1, MEN2A, MEN4, and hereditary hyperparathyroidism-jaw tumor syndrome (HPT-JT) [5].

A small number of cases have reported parathyroid adenoma in patients with FHH [6–13]. In this case report we present a 47-year-old man with severe hypercalcemia, genetic FHH, and a significant parathyroid adenoma at the lower left gland, without clinically actionable variants in *MEN1*.

## Case presentation

In May 2017 the Caucasian patient was admitted to an endocrine clinic due to 6 months of nonspecific symptoms. The patient suffered from general malaise, nonspecific abdominal pain, and paresthesia in his fingers. He had a known history of FHH type 1 which was genetically confirmed in 2008, identifying the pathogenic (class 5) heterozygous *CASR* variant (NM_000388, c.644A>G, p.Asp215Gly in exon 4). The patient owned a medium-sized service company. He lived in the countryside with his wife and children. He had never smoked. At first admission, the patient had no signs of kidney stones and received no treatment. Biochemical evaluation showed elevated plasma ionized calcium (iCa) of 1.87 mmol/L and PTH 17.0 pmol/L with low 25-hydroxy vitamin D (25OHD) of 21 nmol/L and phosphate 0.60 mmol/L. The kidney function was normal, and the initial parathyroid scintigraphy revealed no focus (Table 1). The results of clinical evaluation were normal, with height of 182 cm, weight 83 kg, blood pressure 130/82, heart rate 48 beats per minute, and normal electrocardiography (ECG.) No signs of musculoskeletal symptoms and no neurological symptoms were observed.

**Table 1:** Paraclinical results

Year	Reference value	2008	2017	2018	2019	2019	2019	2019	2019	2019	2020	2021
Month (date)			Oct	June	Jan	Feb	Sept 16	Sept 17	Sept 27	Dec	May	Feb
DNA		*CASR*, *MEN1* (VUS2)							Reevaluation			
Ionized Ca^2+^ (mmol/L)	1.15–1.35	1.60	1.88	1.77	1.89	1.71	1.55	1.45	1.51	1.54	1.58	1.53
Phosphate (mmol/L)	0.71–1.53			0.64	0.52	0.72			0.57	0.57	0.71	0.60
PTH (pmol/L)	1.2–8.3	7.2	12.7	22.2	18.5	6.0			3.8	7.8	6.2	6.3
Vitamin D (nmol/L)	50–160	30	88	46	57	69			64	64	69	74
Hemoglobin (mmol/L)	8.3–10.5		9.6		9.9	9.4			8.9	8.9	9.0	9.2
Creatinine (µmol/L)	60–105		87	86	91	88			80	83	85	85
ALP (U/L)	35–105		99	94	92	85			90	88	76	77
CCCR				0.006								
Cinacalcet (mg)			0	0	120	120	0	0	0	0		0
Parathyroid scintigraphy		No focus				Left lower						
DXA						Normal BMD						
Surgery (pathology)							Adenoma left lower					

*PTH* parathyroid hormone, *ALP* alkaline phosphatase, *CCCR* calcium–creatinine clearance ratio, *DXA* dual-energy X-ray absorptiometry, *BMD* bone mineral density

After 5 months of treatment with 50 µg vitamin D daily, 25OHD had normalized to 88 nmol/L and PTH had decreased to 12.5 pmol/L. However, the symptoms of tiredness, abdominal pain, and hypercalcemia of iCa 1.88 mmol/L remained unchanged. An abdominal computed tomography (CT) scan showed chronic pancreatitis but no renal calcification. The patient was observed (watchful waiting) until June 2018, with no change in clinical symptoms during this period.

In June 2018, reevaluation of the patient showed iCa 1.77 mmol/L, PTH 22.2 pmol/L with 25OHD of 46 nmol/L, and phosphate 0.64 mmol/L. The calcium–creatinine clearance ratio was low, 0.006. All other biochemical measures were normal, including the kidney function.

**Fig. 1:**
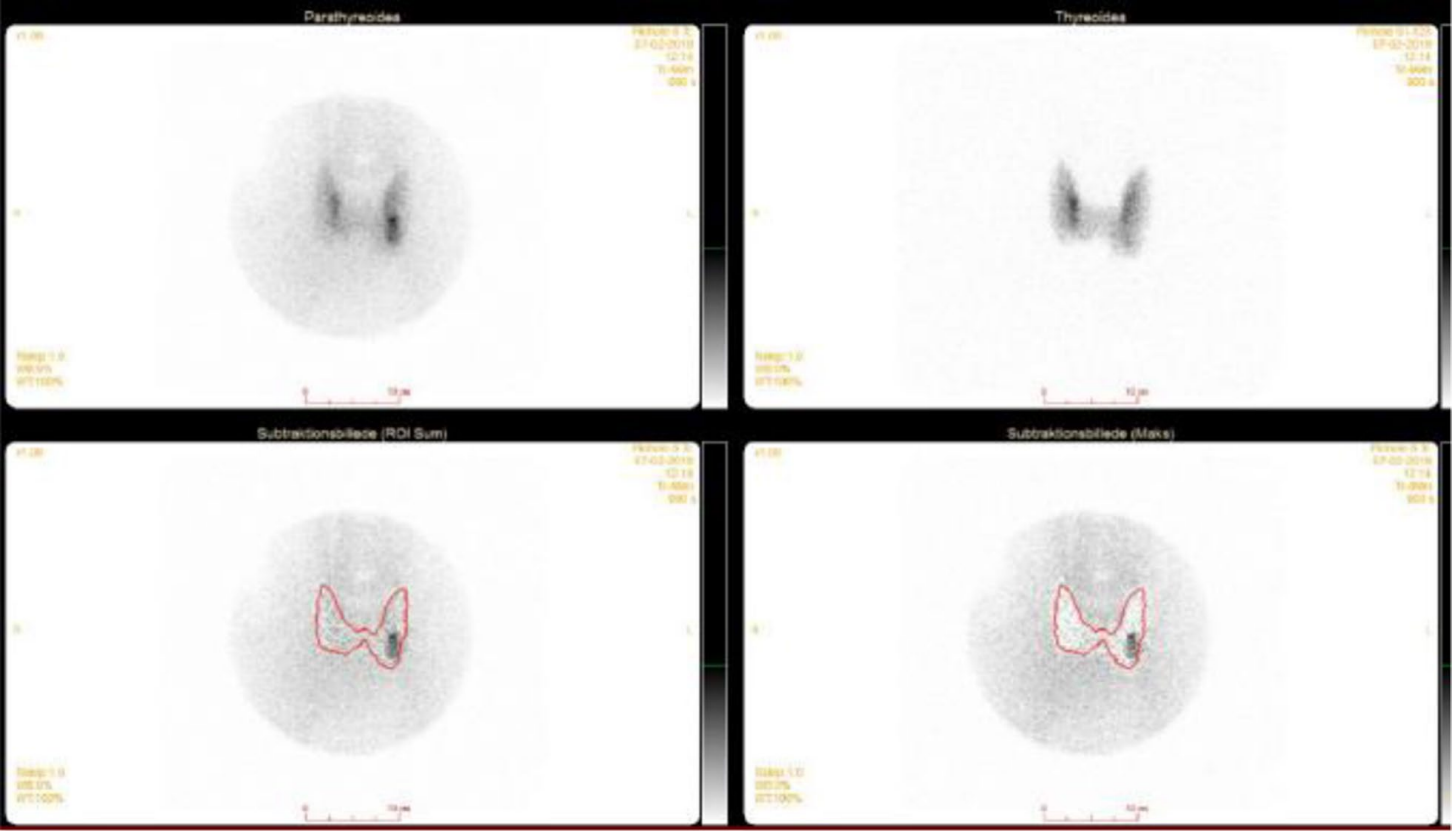
Parathyroid scintigraphy from February 2019 showing a single adenoma at the lower left gland

Three months later, cinacalcet treatment of 60 mg per day was initiated due to persisting high iCa and PTH levels. However, the iCa and PTH levels did not change, and cinacalcet was terminated. In January 2019, cinacalcet was reintroduced due to persistent high levels of iCa of 1.89 mmol/L and PTH of 18.5 pmol/L. A new parathyroid scintigraphy (subtractions-single-photon emission computed tomography/CT) revealed a single adenoma equivalent to the lower left gland corresponding to finding a 1.5-cm adenoma on CT evaluation (Fig. 1).

After a month, the cinacalcet dosage was increased to 60 mg twice daily, as the initial dosage of 60 mg once daily only resulted in a decrease in iCa to 1.71 mmol/L. A dual-energy X-ray absorptiometry (DXA) scan confirmed normal bone mineral density.

After the diagnosis of PHPT was confirmed, parathyroid surgery was completed in September 2019. A single 0.828-g parathyroid adenoma was removed in toto from beneath the left thyroid lobe. PTH decreased 52% perioperatively. The day after surgery, iCa was 1.45 mmol/L. Two weeks post-surgery, iCa was 1.51 mmol/L, and PTH normalized at 3.8 pmol/L. Since surgery, the patient has had no symptoms apart from his chronic pancreatitis. Clinically, the patient showed no hypercalcemic symptoms: blood pressure 121/69, heart rate 50 beats per minute. Respiratory findings were normal, with a rate of 12 breaths per minute and 99% saturation.

Genetic evaluation was redone, adding sequencing of other genes associated with hyperparathyroidism (*AP2S1*, *CASR*, *CDC73*, *CDKN1B*, *GNA11*, *MEN1* and *RET*) including screening for mosaicism. Apart from a likely benign (class 2) *MEN1* variant (c.762 G>A, p.Leu254Leu), no clinically actionable variants were added. The patient was clinically reevaluated 12 month postoperatively with no sign of hypercalcemic symptoms: weight stable at 83.9 kg; height 182 cm; BMI 24.7. Blood pressure was normal at 130/82. ECG was clinically normal with a heart rate of 48 beats per minute; no respiratory pathological findings; no sign of musculoskeletal symptoms; and no neurological symptoms.

**Fig. 2:**
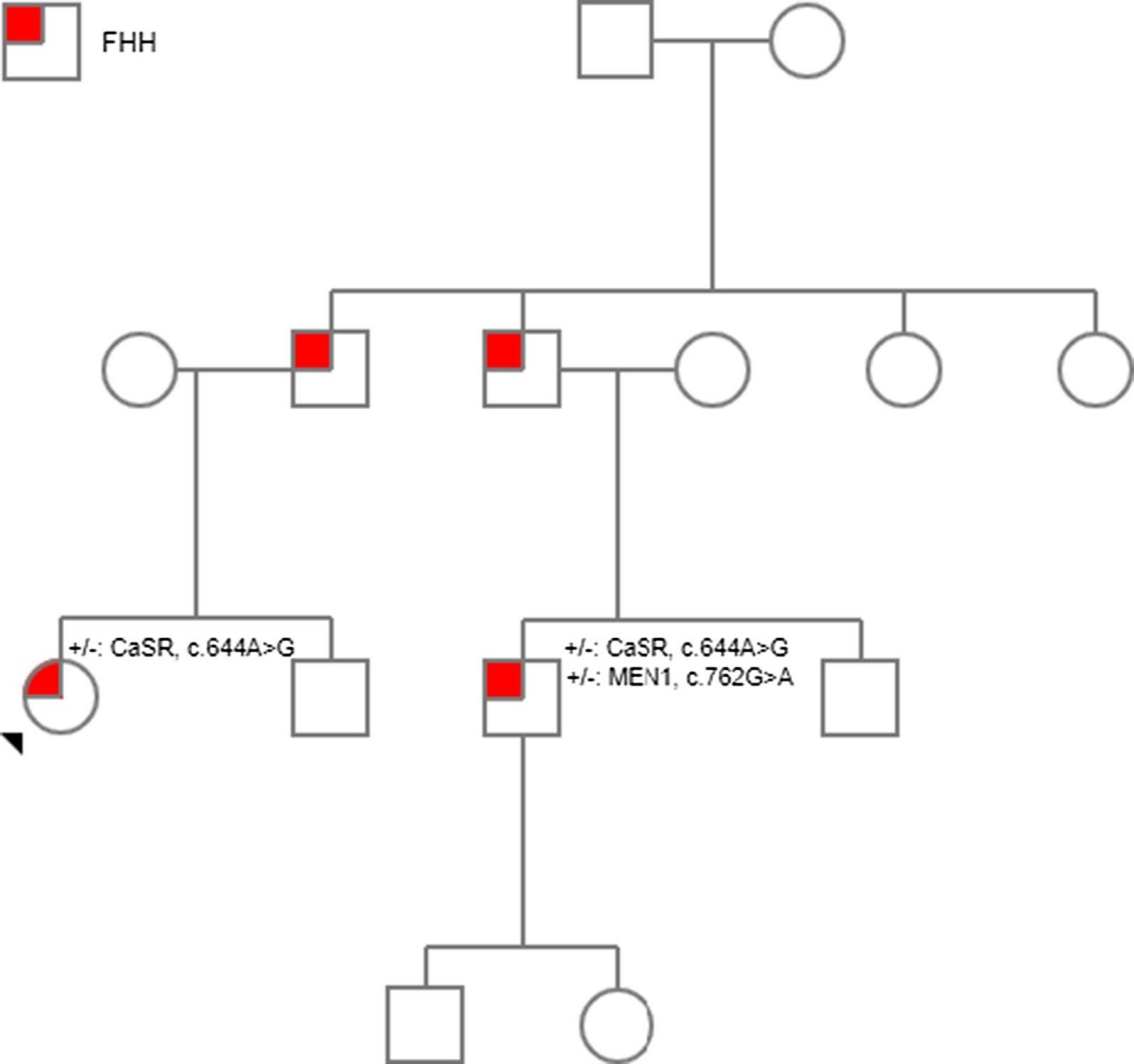
Pedigree of the two related individuals diagnosed with familial hypocalciuric hypercalcemia (FHH). The half-filled symbols indicate the individuals with FHH

In order to confirm that the remaining hypercalcemia was due to FHH, we found that his cousin carried the same *CASR*, c.644A>G, p.Asp215Gly (Fig. 2), and had iCa of 1.44 mmol/L and PTH of 2.1 pmol/L without any other symptoms of disease. The elevated iCa and corresponding normal PTH of this family member confirmed the pathogenicity of the *CASR* variant leading to elevated iCa without hyperparathyroidism.

## Discussion and conclusions

The present case is an example of severe hypercalcemia in a middle-aged man with known FHH due to the development of a parathyroid adenoma. Treatment with high-dose cinacalcet was necessary to lower iCa and improve the patient’s well-being during the diagnostic period. Parathyroidectomy normalized PTH levels and reduced hypercalcemia, and the patient’s hypercalcemic symptoms resolved. PHPT is a common disease in adults. The high parathyroid state in PHPT causes increased renal absorption of calcium, increased bone turnover, and increased activated vitamin D, which in turn enhances calcium absorption from the intestine. PHPT is often asymptomatic and randomly discovered, and only in rare cases can lead to severe life-threatening hypercalcemia. In contrast to PHPT, FHH is a rare, genetic disorder which predominantly results in asymptomatic hypercalcemia with no significant clinical consequences. Our patient had a known pathogenic (class 5) heterozygous *CASR* variant (NM_000388*,* c.644A>G, p.Asp215Gly in exon 4). The variant had previously been identified in another family with FHH, and a functional study had confirmed that the variant leads to inactivation of the protein [14, 15]. The two diseases may share clinical manifestations and biochemical findings as presented in this and other cases. Previous cases of simultaneously diagnosed PHPT and FHH in patients presenting with hypercalcemic symptoms have been described [6–9, 11, 12]. Distinguishing between the two entities can be complicated and must be carefully taken into consideration in the evaluation of hypercalcemia [16]. Reevaluation is necessary if the patient presents residual hypercalcemia after parathyroidectomy. In this case, as in a few others, PHPT develops in a patient with known FHH, which may lead to diagnostic delay, as the clinicians may be more likely to attribute the hypercalcemia to the known disease [10, 13]. Later presentation of hypercalcemic symptoms or organ manifestations, as in our patient developing general malaise and chronic pancreatitis with no obvious cause, should lead to diagnostic reevaluation. Pancreatitis secondary to both PHPT and FHH has been described [17, 18]. The mechanisms, however, are not fully understood. Our patient had persistent nonspecific abdominal symptoms even after parathyroid surgery had restored the calcium levels to FHH baseline levels, probably due to his chronic pancreatitis. These examples of coexisting hypercalcemic disease highlight the importance of following standard diagnostic measures for hypercalcemia as recommended in international guidelines, and reevaluating the patient if changes in clinical or biochemical presentation occur [2, 19]. The consensus of a standard biochemical measure to differentiate between FHH and PHPT is the calcium–creatinine clearance ratio. This measure is not useful for diagnosing concomitant FHH and PHPT, as in the present case. In addition, a recent study showed limitations of the calcium–creatinine clearance ratio in general in differentiating between FHH and PHPT [20]. Thus, a combination of clinical symptoms, family history, biochemical testing, parathyroid scintigraphy, and genetic testing is required to distinguish FHH from PHPT and also to exclude MEN1. In our case, there was a known variant of unknown significance in *MEN1* at the first genetic analysis. As the patient developed PHPT years later, the *MEN1* variant was reevaluated and classified as likely benign. The variant was classified in accordance with American College of Medical Genetics and Genomics (ACMG) guidelines [21]. In conclusion, correct diagnosis of the underlying cause of hypercalcemia is important to ensure the right treatment, as highlighted by the present case of a patient with known FHH developing PHPT. Patients with FHH should avoid operative treatment, and PHPT should be differentiated from MEN1 to determine whether parathyroidectomy should include the removal of one adenoma or 3.5 hyperplastic parathyroid glands.

### Patient perspective

The patient was afraid of malignant parathyroid disease and treatment thereof as he since childhood had been aware of the FHH diagnosis and reassured of the benign genesis.

## Data Availability

All relevant patient data are included in the case report, and further supporting data may be obtained on request.
